# Long-term nitrofurantoin: an analysis of complication awareness, monitoring, and pulmonary injury cases

**DOI:** 10.3399/BJGPO.2021.0083

**Published:** 2021-10-27

**Authors:** Toby Peter Speirs, Nicole Tuffin, Finlay Mundy-Baird, Helena Sakota, Sarah Mulholland, Michelle Westlake, Max Lyon, Andrew R Medford, Charles Sharp, Michael Darby, Mahableshwar Albur, Francis Keeley, Helena Burden, Charlie Kenward, Elizabeth Jonas, Shaney Barratt, Huzaifa I Adamali

**Affiliations:** 1 Bristol Interstitial Lung Disease Service, Southmead Hospital, North Bristol NHS Trust, Bristol, UK; 2 Department of Radiology, North Bristol NHS Trust, Bristol, UK; 3 Department of Microbiology and Infectious Disease, North Bristol NHS Trust, Bristol, UK; 4 Department of Urology, North Bristol NHS Trust, Bristol, UK; 5 NHS Bristol, North Somerset and South Gloucestershire Clinical Commissioning Group, Bristol, United Kingdom

**Keywords:** nitrofurantoin, general practice, urology, drug-related side effects and adverse reactions, prescription drug monitoring programs

## Abstract

**Background:**

Long-term nitrofurantoin (NF) treatment can result in pulmonary and hepatic injury. Current guidelines do not outline the type or frequency of monitoring required for detection of these injuries.

**Aim:**

To assess 1) awareness of NF complications among prescribers; 2) monitoring practice; and 3) to describe the pulmonary sequelae of NF-related complications.

**Design & setting:**

Evaluation of prescribing habits by questionnaires and review of GP databases, and case-note review in secondary care.

**Method:**

The following study procedures were undertaken: 1) an electronic questionnaire was distributed to prescribers, interrogating prescribing and monitoring practices, and awareness of complications; 2) an analysis was undertaken (June–July 2020) of NF monitoring among GPs in the local clinical commissioning group (CCG); and 3) a case review was carried out of patients diagnosed with NF-induced interstitial lung disease (NFILD) at the interstitial lung disease (ILD) centre (2014–2020).

**Results:**

A total of 125 prescribers of long-term NF responded to the questionnaire (82.4% GPs; 12.0% urologists). Many were unaware of the potential for liver (42.4%) and lung (28.0%) complications; 40.8% and 52.8% never monitored for these, respectively. Only 53.3% of urologists believed themselves responsible for arranging monitoring, while nearly all GPs believed this to be the prescriber’s responsibility (94.2%). One-third of all responders considered current *British National Formulary* (*BNF*) guidelines 'not at all sufficient/clear', with mean clarity scoring of 2.2/5. Among patients with NFILD (*n* = 46), NF had been prescribed most often (69.6%) for treatment of recurrent UTI and 58.6% (*n* = 27) were prescribed for >6 months. On withdrawal of the medication 61.4% displayed resolution (completely or minimal fibrosis), while 15.9% of patients had progressive lung fibrosis.

**Conclusion:**

NF can cause marked or irreversible lung complications and there is currently a shortfall in awareness and monitoring. Existing monitoring guidelines should be augmented.

## How this fits in

It is known that long-term NF use can induce hepatic and/or pulmonary complications, and that monitoring is required for early detection. This research highlights a shortfall in 1) awareness of NF-related side effects and 2) monitoring practice in the prescribing community. Hence, the following points are suggested: awareness of potential NF complications should be raised among GPs and urologists; monitoring responsibilities need to be clarified; and existing monitoring guidelines should be augmented.

## Introduction

NF is prescribed for treatment of acute urinary tract infections (UTIs) and prophylactically to reduce UTI recurrence.^1,2^ Complications, notably hepatic and pulmonary, have been associated with short-term and long-term use and are more common in women.^3–5^ NFILD refers to a spectrum of lung changes, from an acute hypersensitivity reaction (within 1–2 weeks) to a chronic pulmonary reaction involving fibrosis (months to years of exposure). The prevalence of severe adverse events was estimated at 0.2% (95% confidence interval [CI] = <0.01% to 1.2%) in controlled trials and from 0.02 to 1.5 events per 1000 NF users in observational studies.^6^ Although severe adverse events may be uncommon in a general population, the risk increases with longer treatment durations and the reported prevalence among individuals aged ≥65 years is 2.1%.^5^ The precise mechanisms of adverse effects are not fully understood.^7–9^

Current adult *BNF* guidelines state: *'* *On long-term therapy, monitor liver function and monitor for pulmonary symptoms, especially in the elderly (discontinue if deterioration in lung function)* *.’* ^10^ However, neither a method nor frequency of hepatic or pulmonary monitoring, nor a definition for 'long term' is suggested. National Institute for Health and Care Excellence (NICE) guidance on antimicrobial prescribing for recurrent UTI recommend review at 6 months.^11^ Both NICE guidance and local CCG guidelines rely on referencing *BNF* advice.^2^

Negligence in NF monitoring has been highlighted as a significant cause of litigation, according to the UK Medical Protection Society (UKMPS)^12^ that advises liver function testing (LFT) and reviews for respiratory symptoms at least 6-monthly, with consideration of more frequent monitoring.^12^

Improving monitoring protocols may speed up recognition of complications and withdrawal of NF to lessen severity of toxicity. The current landscape of monitoring practice is unknown. This study aimed to determine: 1) the awareness of NF side effects; 2) the existing monitoring practices of NF in general practice within the local area; and 3) to describe the cohort of patients diagnosed with NFILD by multidisciplinary team (MDT) consensus at the Bristol Interstitial Lung Disease (BILD) service.

## Method

### Setting

This study involved collaboration of the BILD service at North Bristol NHS Trust (NBT) and the Bristol, North Somerset and South Gloucestershire (BNSSG) CCG. The BILD service provides secondary and tertiary specialist ILD care to a catchment of 1.2 million in South West England.

### Study procedures

#### Questionnaire: assessing prescribing insight

A 13-item questionnaire was electronically distributed (July 2020) to all local GPs (*n* = 675), nurses (*n* = 145), CCG medicine optimisation pharmacists (MOPs; *n* = 72), and South West urologists (*n* = 130) (see Supplementary Appendix S1). Responses were anonymised to ensure confidentiality and encourage candid responses.

#### Analysis of monitoring practices of NF within primary care

Data were requested from all CCG GP practices (*n* = 78), collated by MOPs, and submitted to study organisers for analysis. Monitoring history was analysed for all patients with an active NF prescription at the time of data collection (26 June–25 July 2020) and registered at a participating GP practice. Patient demographics and extent of NF exposure (dose and duration: episodic, continuous, or cyclical use) were collected from GP practice records. Data regarding relevant monitoring procedures performed at baseline and subsequently were also collated, including clinical examination, oxygen saturation, chest X-ray (CXR), spirometry, and LFTs. Only patients with a NF prescription initiated within the preceding 2 years were included, to address contemporary practice.

#### Case-note review and characterisation of patients with an MDT consensus diagnosis of NFILD

Patients with an MDT consensus diagnosis of NFILD (September 2015–July 2020) were retrospectively identified from the BILD database. From records of patients diagnosed with NFILD, data were collated regarding basic demographics and NF exposure. Baseline (initial presentation) and final (completion of audit) lung function were documented. Spirometry measurements included forced vital capacity (FVC), forced expiratory volume in 1 second (FEV1), diffusion capacity of the lungs (TLCO), and TLCO with correction for haemoglobin (KCO). Six-minute walking distance (6MWT), oxygen saturations, and MRC dyspnoea scores were also compared between initial and final visits. Baseline and final high-resolution computerised tomography (HRCT) imaging results were independently reviewed for changes to radiological pattern by a thoracic radiologist and a respiratory consultant. Treatment regimens including long-term or ambulatory oxygen therapy (LTOT or AOT) and steroids were interrogated.

### Statistical analysis

Continuous variables were tested for normality using the D’Agostino-Pearson test. Where parametric, data were tested by paired two-tailed *t*-tests and presented as mean±standard deviation (SD). Where non-parametric, data were tested by Wilcoxon tests (paired) or Mann-Whitney U tests (unpaired) and presented as medians with interquartile range (IQR). For all statistical tests, *P*≤0.05 was considered statistically significant. Data were analysed using GraphPad Prism 8 for Windows (version 8.4.3).

## Results

### Questionnaire: assessing prescribing insight

Response rate among GPs and urologists, respectively, was 35.9% (*n* = 242) and 31.5% (*n* = 41). Long-term (>6 months) NF was prescribed by 39.8% (*n* = 125) of the 314 total responders: GPs 82.4% (*n* = 103), urologists 12.0% (*n* = 15).

CCG guidelines were preferred (47.2%, *n* = 59), followed by NICE (38.4%, *n* = 48), and local trust guidelines (10.4%, *n* = 13). The questionnaire also found 11.2% (*n* = 14) did not use guidelines and 65.6% (*n* = 82) of prescriptions were for recurrent UTIs.

For treatment of recurrent UTIs, most prescribers based their antibiotic choice on culture results (52.8%, *n* = 66). However, the first-choice treatment was NF for 28.0% (*n* = 35) and trimethoprim for 18.4% (*n* = 23).

Many prescribers were unaware of the potential hepatotoxicity (42.4%, *n* = 53) or pulmonary toxicity (28.0%, *n* = 35). In addition, 52.8% (*n* = 66) of prescribers reported that they did not measure baseline function at prescription (respiratory symptoms, oxygen saturation, respiratory examination, CXR, spirometry, and LFTs). The most common baseline test or examination was LFT (32.8%, *n* = 41), while 20.8% (*n* = 26) documented baseline respiratory symptoms. A minority (<5%) checked other modalities (oxygen saturations, respiratory examination, CXR, and spirometry) and some mentioned taking a history of previous lung and/or liver abnormalities and blood tests to assess for infection.

**Figure 1.**
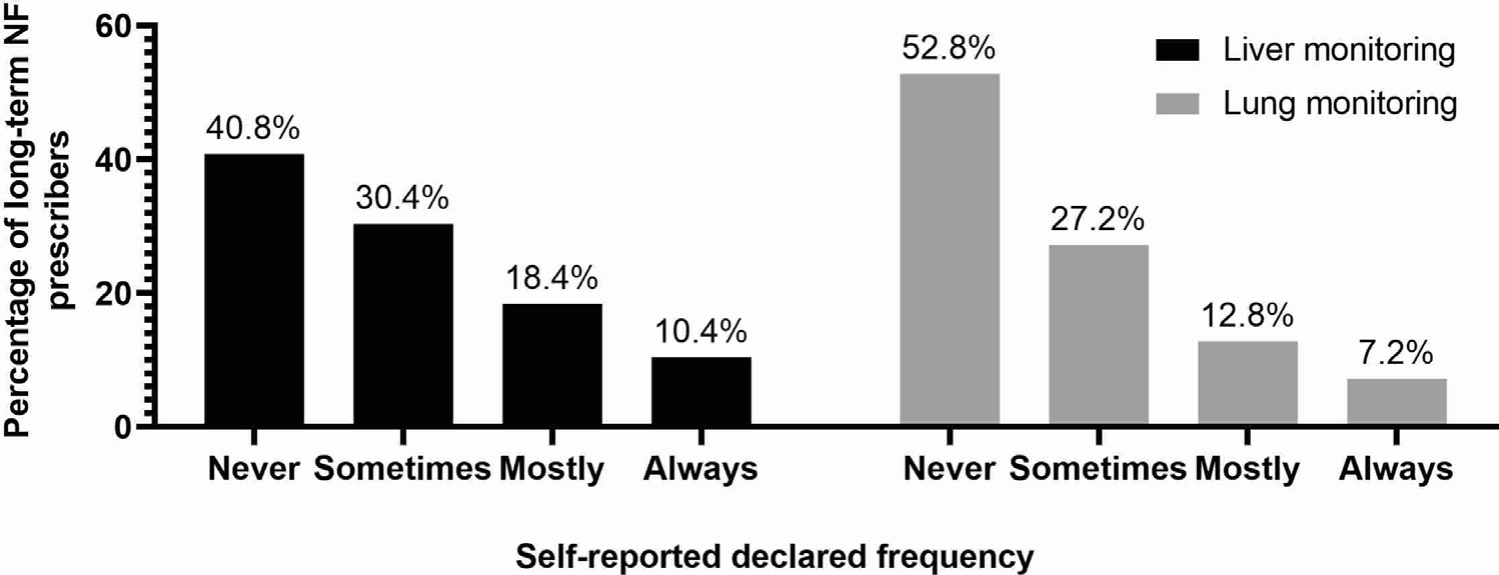
Frequency with which long-term prescribers of NF self-reported initiating a monitoring programme for patients using NF. NF = nitrofurantoin.

The questionnaire investigated frequency of follow-up monitoring. LFT was reportedly 'always' monitored by 10.4% (*n* = 13) and 'lung-related examination and tests (LuRT)' 'always' by 7.2% (*n* = 9) of prescribers. The most common tendency was to 'never' monitor liver (40.8%, *n* = 51) or lung function (52.8%, *n* = 66) (Figure 1). When prescribers did monitor, this was most commonly performed every 12 months (32.0%, *n* = 40).

The questionnaire gauged agreement with this statement: *'* *T* *he prescriber of nitrofurantoin is responsible for monitoring the drug side effects/complications* *.'* Nearly all GPs (94.2%, *n* = 97) considered monitoring to be the responsibility of the prescriber in contrast to 53.3% (*n* = 8) of urologists.

**Figure 2.**
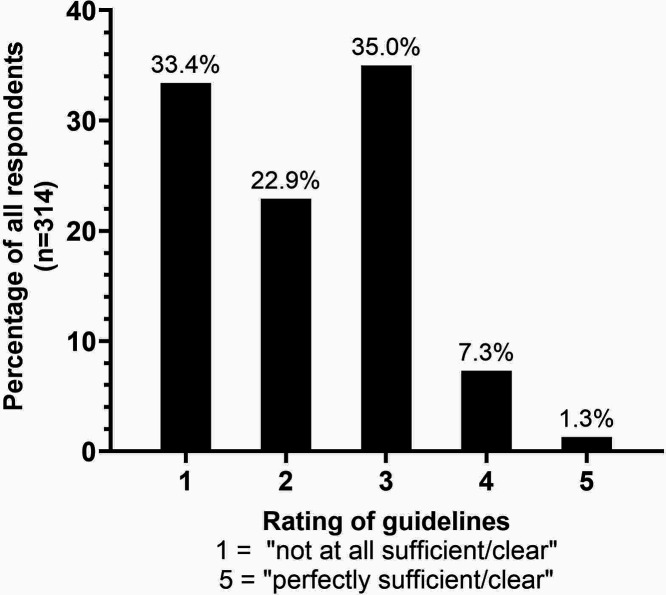
Reported quality rating of current prescription guidelines for NF, across all responders of the questionnaire. NF = nitrofurantoin.

The questionnaire interrogated the perceived clarity and sufficiency of current guidelines for long-term NF prescription. One-third of responders (*n* = 105) considered guidelines to be *'* *not at all sufficient/clear* *'* whereas 1.3% (*n* = 4) considered them to be *'* *perfectly sufficient/clear* *'* (Figure 2). The mean rating of existing guidelines was 2.2/5 (SD 1.0).

### Analysis of monitoring practices of NF within primary care

Data were collated from 62 of 78 GP practices in the CCG (79.5%). A total of 503 patients used long-term NF (July 2020). Those with NF prescription initiated within the preceding 2 years are considered herein (*n* = 265). The cohort was predominantly female (81.5%, *n* = 216) and aged >60 years (Table 1).

**Table 1.** Age distribution of the 503 patients prescribed long-term (>6 months) NF at GP practices (*n* = 78) within the BNSSG CCG.

Age range, years	**%**
0–20	3.0
21–40	18.1
41–60	25.3
61–80	38.1
≥81	15.5

BNSSG CCG = Bristol, North Somerset and South Gloucestershire clinical commissioning group. NF = nitrofurantoin.

Baseline monitoring (tests undertaken at prescription date±1 month) of any kind was absent in 73.6% (*n* = 195) of patients; 11.7% (*n* = 31) received only LFTs and 10.2% (*n* = 27) only LuRT at baseline. The remaining minority (4.5%, *n* = 12) had both LFT and LuRT at baseline.

A subcohort of patients (39.6%, *n* = 105) had been on NF for 6–24 months. Of these, many received no monitoring beyond 6 months post-prescription (44.8%, *n* = 47), had only LFT monitoring (20.0%, *n* = 21), or had only LuRT monitoring (14.3%, *n* = 15). Relatively few patients received both liver and lung monitoring (21.0%, *n* = 22).

### Case-note review and characterisation of patients with an MDT consensus diagnosis of NFILD

The BILD service database archived 16 472 MDT discussion cases of 10 500 patients (January 2014–June 2020). Of these, a consensus diagnosis of NFILD was made in 46 patients (September 2015–June 2020) (0.4% of patients).

The median age was 72.0 years (IQR 66.3–78.8, range 34–91). The majority were female (80.4%, *n* = 37). Baseline characteristics are summarised in Table 2. The major indications for NF were as a prophylactic treatment for recurrent UTIs of unspecified cause (69.6%, *n* = 32) and catheter-related recurrent UTIs (10.9%, *n* = 5). Other indications included UTI and recurrent cystitis.

**Table 2.** Baseline characteristics of BILD patients with NF-induced interstitial lung disease (NFILD).

**Characteristic**	**Mean** **±SD^a^**
Male:female ratio	9:37
Age, years	71.76±1.62
**Lung Function**	
FVC (L)	2.30±0.11
FEV1 (L)	1.82±0.07
Ratio (%)	79.8±1.43
TLCO (mmol/min/kPa)	4.10±0.23
KCO (mmol/(min/kPa/L)	1.27±0.08
**Six** **-** **m** **inute** **w** **alk** **t** **est**	
Distance walked (m)	270.3±22.8
Minimum desaturation (%)	89.5±0.87
MRC dyspnoea score	3.3±0.17

^a^Unless otherwise stated. BILD = Bristol Interstitial Lung Disease. FEV1 = forced expiratory volume in 1 second. FVC = forced vital capacity. KCO = TLCO correction for haemoglobin. MRC = Medical Research Council. TLCO = diffusion capacity of the lungs.

The duration of NF exposure preceding toxicity ranged from <1 week (acute adverse effect) to ≥12 months. Exposure duration was 6–12 months for 19.6% (*n* = 9) and ≥12 months for 39.1% (*n* = 18). Duration was unknown for 32.6% (*n* = 15) of patients.

**Figure 3.**
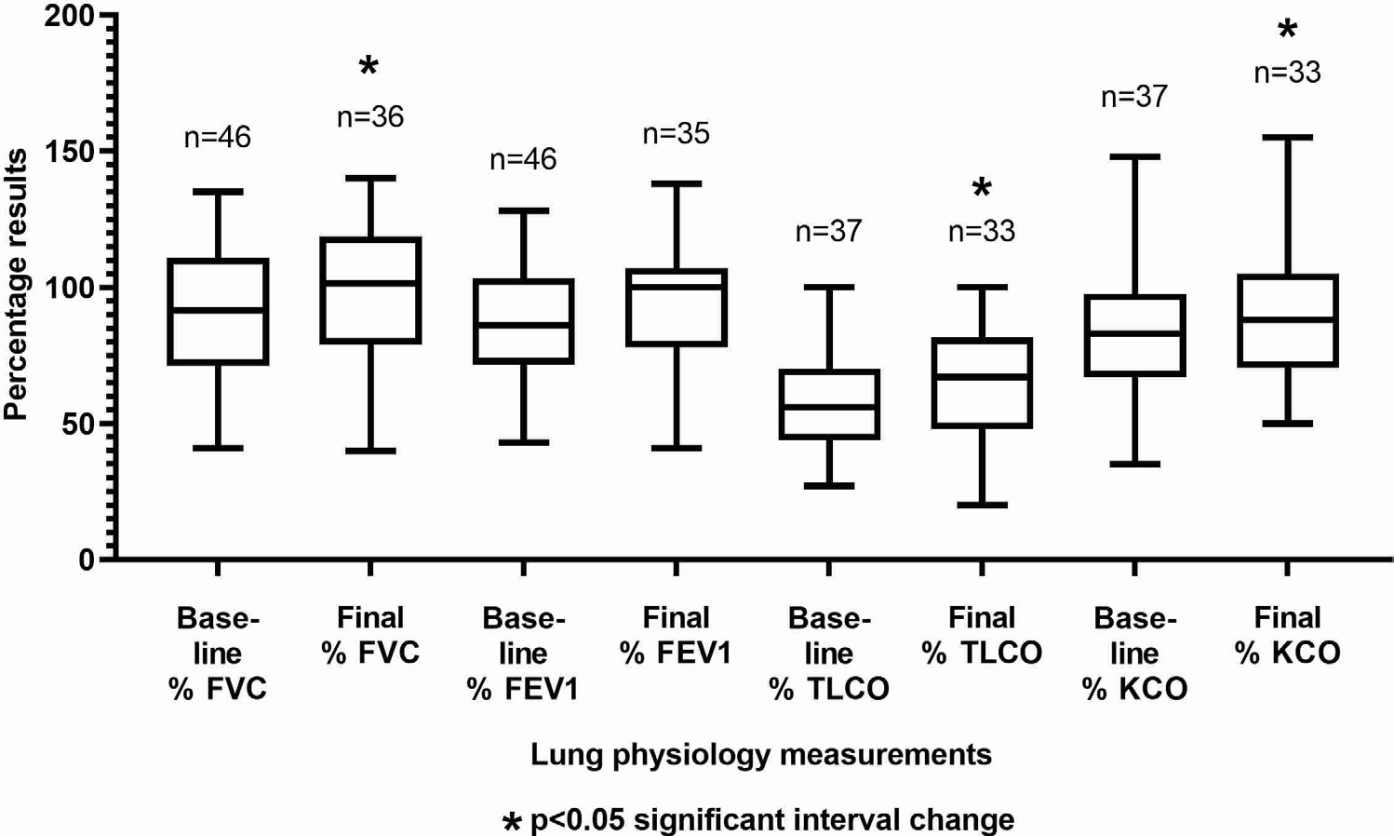
Spirometry test result changes over interval of Bristol Interstitial Lung Disease clinic treatment for nitrofurantoin-induced interstitial lung disease. FVC increased by median 5.00% (95% CI = –0.79 to 15.18). FEV1 increased by mean 2.93% (95% CI = –4.86 to 10.72). TLCO increased by mean 7.70% (95% CI = 2.49 to 12.91). KCO increased by mean 7.10% (95% CI = 2.27 to 11.94). FEV1= forced expiratory volume in 1 second. FVC = forced vital capacity. KCO = TLCO correction for haemoglobin. TLCO = diffusion capacity of the lungs.

The median follow-up period by the BILD service following NF cessation was 11.0 months (IQR 6.0–23.0). From baseline to final clinic visit, spirometry showed statistically significant improvements in FVC (*P* = 0.041), TLCO (*P* = 0.005), and KCO (*P* = 0.006) (Figure 3).

There was no significant change in resting oxygen saturation (mean 95.4±2.1% versus 96.1±1.7%, *P* = 0.68), 6MWT (median [IQR] absolute distances 280.0 m [211.3–345.5] versus 280.0 m [220.0–320.0], *P* = 0.14), or desaturation during 6MWT (median [IQR] 90.0% [86.8–93.0] versus 92.5% [91.0–93.0], *P* = 0.34) between initial visit and final visits. Notably, there was significant improvement in MRC dyspnoea score (mean 3.3±1.2 versus 2.9±1.3, *P* = 0.011).

The patterns of fibrosis at baseline HRCT included cellular non-specific interstitial pneumonia (NSIP) (13.0%, *n* = 6), fibrotic NSIP (17.4%, *n* = 8), organising pneumonia (OP) (10.9%, *n* = 5), fibrotic OP (2.2%, *n* = 1), and hypersensitivity pneumonitis (13.0%, *n* = 6), with an overlap of features in the remaining 20 patients.

**Figure 4.**
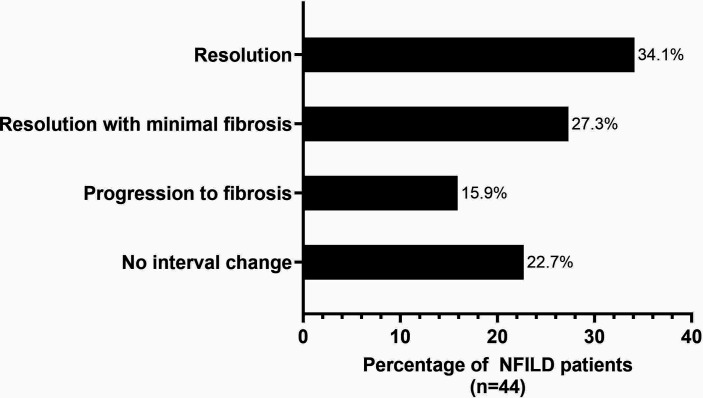
Interval radiological outcomes of patients with NFILD followed-up in the BILD clinic

Follow-up HRCT were available in 44 patients, at a median interval of 10.5 months (IQR 5.0–22.0). Interval radiological changes (Figure 4) in most cases showed complete resolution or minimal fibrosis (61.4%, *n* = 27). Some showed progression of fibrosis over the follow-up period (15.9%, *n* = 7). The remainder of patients showed no change (22.7%, *n* = 10).

HRCT radiological outcomes were not statistically significant between patients who were corticosteroid-treated and those who had not received corticosteroids (*n* = 29 and *n* = 15, respectively, *P* = 0.83), although the cohorts were small.

Of the 46 patients, four consequently required LTOT and five required AOT.

## Discussion

### Summary

This study has found low awareness of complications and suboptimal monitoring associated with long-term NF treatment, evident from both prescriber self-reporting and patient records. The authors advocate augmentation of current guidelines with a model monitoring plan. The pulmonary outcomes of patients with NFILD serve as a warning of some potential consequences of low awareness and monitoring, even before exploring hepatic impacts.

Inadequate monitoring delays drug cessation and increases toxicity.^6^ The questionnaire revealed low rates of active baseline testing, required to recognise any abnormalities in future monitoring results. However, in practice, many patients may have received 'passive' baseline testing associated with routine health checks. By the authors' definition of baseline monitoring, it has been shown that monitoring in practice may be lower than self-reported monitoring. Follow-up monitoring is also low; large proportions of prescribers reported that they 'never' monitor liver or lung complications, and only a minority 'always' monitor these.

The questionnaire also uncovered a discrepancy in perceived responsibilities between primary and secondary care professionals. Some primary care responders expect secondary professionals to perform baseline tests. Furthermore, almost all GPs, compared with approximately half of urologists, considered monitoring to be the prescriber’s responsibility. Effective communication between primary and secondary care is vital to ensure high-quality health care. Breakdown of this communication can result in reduced quality of care.^13^

### Strengths and limitations

This study engaged all parties involved in prescribing and monitoring of NF. The questionnaire had an adequate response rate from a large sample population, but remains susceptible to response bias. Despite this, among responders, the results are concerning and suggest a general low awareness and/or perceived significance of the problem. All LFTs in data collection were considered as undertaken for monitoring purposes. It is not possible to attribute this with certainty; many LFTs are likely to be coincidental given the cohort’s advanced age and comorbidities. Hence, deliberate hepatic monitoring was likely overestimated. It should also be acknowledged that GPs often rely on existing clinical information and/or results in decision making about baseline safety for prescribing.

It is recognised that the sample of patients with NFILD was relatively small. Nonetheless, the primary focus of this study was monitoring, not sequelae of complications. There are further limitations with retrospective study that include missing data. The COVID-19 pandemic may have affected the final 4 months of the investigation window, possibly impacting follow-up, treatment routines, or respiratory outcomes for some patients.

### Comparison with existing literature

Although complications of NF have long been recognised,^14,15^ there is a dearth of studies on awareness and/or monitoring for NF complications. Audits examining other medications with known potential pulmonary side effects show a similar monitoring shortfall.^16–19^

Other large NF-related studies have shown similar pulmonary complications.^7,15,20–22^ Demographics of these larger cohorts also matched this cohort in age distribution and sex ratio.^4,5,23^

A small proportion of patients with NFILD demonstrated progressive fibrosis despite cessation. Current literature has raised concerns about irreversible pulmonary sequelae of NF use,^8,21,23–25^ although concurrent ILD diagnosis unrelated to NF cannot be excluded. Most of the cohort were exposed to NF for >6 months before complications. Comparing radiological outcomes and duration of exposure presented evidence of a positive correlation between duration and chronic impacts, consistent with Holmberg and colleagues’ findings; 47% of their cohort with chronic respiratory disease developed this following at least 12 months of NF.^3^

### Implications for research and practice

Progressive ILD is a significant outcome from long-term NF administration, as well as being a medicolegal prescribing risk. This warrants improvements in baseline assessment and monitoring of patients prescribed NF. Development of education and explicit guidelines on the risks of NF complications could address the low prescriber awareness highlighted in this study and improve compliance. GPs are urged when prescribing NF to be wary of its hepatic and pulmonary risks.

Most questionnaire responders used CCG and NICE guidelines for monitoring and rated current guidelines poorly (2.2/5). *BNF* guidelines state that prescribers should *'* *monitor liver function and monitor for pulmonary symptoms* *'* ^10^ but there is no reference to baseline testing, nor a suggested monitoring plan. Such guidelines should be augmented, emphasising risks, and detailing monitoring plans.

Novel guidance may include but is not limited to:

1. Clear verbal and written information for patients defining potential side-effects of NF, documenting this provision;
2. Need for baseline oxygen saturations, MRC dyspnoea score, respiratory examination, ideally CXR in last 6 months, and LFT and creatinine clearance in last 12 months;
3. Need for regular follow-up monitoring: respiratory symptoms, oxygen saturations, MRC dyspnoea score, respiratory examination and LFT, ideally at 3-monthly periods during dosing;
4. Rapid cessation if toxicity is suspected;
5. Rapid accessibility to local hepatology and respiratory services; and
6. Review of automated warning and/or monitoring signals on electronic prescribing platforms.

Spirometry has been deliberately excluded given the challenge of delivering this in the current pandemic. This guidance may be sufficient to detect medication-related toxicity. The authors recognise that this guidance may represent an increased burden on GPs. Before any guidance is finalised, a cost-effectiveness analysis could be undertaken, potentially including a modelling study to ascertain the optimal set of monitoring items, hence determining the most effective way to mitigate risk while recognising feasibility.

A further safeguard is improvement of communication pathways between GPs and urologists, so that all parties are aware that the prescriber is responsible for monitoring. Novel guidance could clarify such recommended communication.

The majority of patients registered at BILD were exposed to NF for >6 months before complications. There is need for further research into causative durations, but limiting use to 6 months and/or cycling it with a different antibiotic may prove protective.^26^ Beyond 6 months, clinicians should reconsider the prescription in light of its risks.
